# The integrated information Φ of an integrate and fire network

**DOI:** 10.1371/journal.pcbi.1014085

**Published:** 2026-03-09

**Authors:** Miłosz Danilczuk, Marek Pokropski, Piotr Suffczynski

**Affiliations:** 1 Faculty of Physics, University of Warsaw, Warsaw, Poland; 2 Faculty of Philosophy, University of Warsaw, Warsaw, Poland; Ben-Gurion University of the Negev, ISRAEL

## Abstract

Integrated Information Theory is a theoretical framework proposing that consciousness is a fundamental property of systems capable of integrating information. To bridge the gap between the theoretical concept and the practical use in actual neurobiological systems, we have applied the Integrated Information Theory approach to a simulated network of integrate and fire neurons (IAF). The primary contribution of this study is several empirical findings. Our analysis shows that such a network can possess a non-zero Φ value under certain conditions and parameter settings. Additionally, our research indicates that the complexity of the network’s dynamics doesn’t necessarily correlate with its Φ value. On the other hand, the quantity of integrated information within the network appears to grow with the IAF neurons’ time constant, which reflects their integrative capacity. Furthermore, our examination of the integrate and fire network with internal random fluctuations demonstrates that the integrated information measure, as defined in IIT version 3.0, is not resilient to noise.

## Author summary

In this study, we explored how Integrated Information Theory (IIT), one of the leading theories of consciousness, can be applied to a small network of simulated neurons. Specifically, we used a network of artificial integrate-and-fire neurons that mimic some properties of real ones to test how much information such a network can integrate, which IIT quantifies using a value called Φ. Our findings indicate that under certain conditions, these networks can indeed integrate information and produce non-zero Φ values. However, we also discovered that the network’s complexity doesn’t necessarily correlate with the amount of information it integrates. Instead, the ability of neurons to retain information over time (their time constant) appears to have a more significant impact. Importantly, we also found that the integrated information measure is sensitive to noise—random fluctuations that are always present in real brains. This suggests that the version of IIT used in our study is responsive to the noisy nature of biological systems. Overall, our study presents several empirical findings on testing the IIT framework in a simple neural system and underscores the key limitations of applying this theory to realistic neuronal models.

## Introduction

Integrated Information Theory (IIT) is a prominent and highly discussed theory of consciousness introduced by Giulio Tononi and others in the late 2000s and since then it has been continuously developed (in this article, we refer to IIT version 3.0 described in [1–3]. The theory is based on the idea that consciousness arises from integrated information within a complex system, such as the brain [4]. IIT adopts a top-down theoretical approach, rather than a bottom-up brain-centered approach to explaining consciousness. This means that IIT begins from phenomenological axioms, i.e., key assumptions about our conscious experience, and then infers from them postulates concerning the properties of underlying physical mechanisms, i.e., necessary conditions that a physical system must fulfill to support consciousness.

According to IIT, there are five phenomenological axioms that grasp the fundamental properties of our experience: intrinsic existence (experience exists from an intrinsic perspective), composition (experience is composed of phenomenological distinctions), information (conscious experiences are informative, i.e., a particular experience is different from any other possible experience), integration (experience is unified, i.e., it is not reducible to its phenomenological components), and exclusion (experience is definite in its contents and spatio-temporal grain) [2]. Integration and exclusion are particularly important axioms since they provide the way to measure the level of consciousness in a physical system. Postulate derived from the axiom of integration states that the system’s cause-effect structure must be unified, i.e., it is “intrinsically irreducible to that specified by non-interdependent sub-systems obtained by unidirectional partitions” [2]. The measure of such an intrinsic irreducibility is integrated information. From the axiom of exclusion, we infer that the system’s cause-effect structure must be definite, this means that it is specified by a single set of elements over which it is maximally irreducible. Such a maximally irreducible cause-effect structure is called a conceptual structure, which, according to IIT, is identical to the system’s content of conscious experience [2].

One of the merits of IIT is that it provides a sophisticated way to quantify the amount of integrated information within a system, a measure called Φ. Respectively, the amount of information integrated in the maximally irreducible cause-effect structure is expressed by Φ^max^, which can be understood as a proxy for the level of consciousness in the target system. If Φ^max^ equals 0, then the conceptual structure is fully reducible to its parts and the system is not conscious. If the value of Φ^max^ is above zero, then the system has a certain degree of consciousness. A consequence of this theoretical and mathematical approach to consciousness is that consciousness is a gradable phenomenon and that, in principle, it can be ascribed to any physical system for which Φ^max^ is a non-zero value.

IIT has recently been the subject of strong criticism. It was criticized for implying a limited form of panpsychism, e.g., [5]. Some researchers also have objections to the proposed set of axioms and their self-evident or irrefutable nature, e.g., [6,7]. Others have argued that there are problems with the testability of IIT’s predictions, e.g., [8]. In this article, we address another issue with IIT, namely that the application of IIT to continuous real-world systems is limited. Only a few studies have so far explored information structures in non-neuronal biological systems [9,10] and in the human brain [11].

The IIT framework is typically applicable to discrete Markovian dynamical systems that satisfy the conditional independence property [12]. A Markovian dynamical system is a type of dynamical system where the future state of the system depends only on its present state and not on the past states that led to it. In Markov processes, the conditional independence property means that once we know the present state, the past states do not affect predictions of the future states. So far, IIT’s approach has been applied to “toy” systems, including abstract networks composed of logic gates [1], systems inspired by Process Algebra [13], interacting units driven by Gaussian linear autoregressive dynamics [14], or networks featuring logical variables with multiple values [15], all satisfying the conditions of Markovian systems. In contrast, biological neurons may exhibit non-Markovian behavior due to long-term dependencies related to ion channel kinetics [16] and history-dependent synaptic plasticity [17]. Nevertheless, single neurons and networks of neurons can often be modeled as Markovian dynamical systems under certain assumptions, especially in simplified mathematical models [18,19]. Here, we demonstrate that IIT can be applied to a simulated network of integrate and fire (IAF) neurons, which are commonly used to model brain functions [20]. Our analysis shows that such a network may be characterized by a non-zero Φ value. Additionally, we demonstrate that the amount of integrated information in the network tends to increase with the IAF neuron time constant, which reflects its integrative capacity.

## Results

### Approach evaluation

First, we verified the correctness of our Transition Probability Matrix (TMP) calculation and IAF neuron implementation. This was achieved by emulating the logic gate system with the IAF network and comparing the numerical values of Φ from a simulated network to those obtained from the logic gate system described in [12]. We confirmed that the IAF neurons with membrane time constant equal to a single time-step, i.e., *τ* = 1*Δt*, all weights equal to 50 mV, *V*_*rest*_ = 0 mV, and appropriately selected threshold values (see Methods) successfully emulated logic gates and produced Φ values closely matching results for the ABC system provided in the PyPhi visual interface described in [12] (Table 1).

**Table 1:** Comparison of Φ values produced by the visual interface described in [12] (second column) and obtained with a network of IAF neurons (third column).

State (A, B, C)	Φ visual interface	Φ of IAF network
(0,0,0)	0.66667	0.666668
(0,0,1)	0.25	0.25
(0,1,0)	‘The selected subsystem is in an impossible state’	‘unreachable’
(0,1,1)	‘The selected subsystem is in an impossible state’	‘unreachable’
(1,0,0)	1.91667	1.916665
(1,0,1)	1.81667	1.816667
(1,1,0)	0.25	0.25
(1,1,1)	0.66667	0.666668

### Tracking the IAF network activity in time

**Fig 1:**
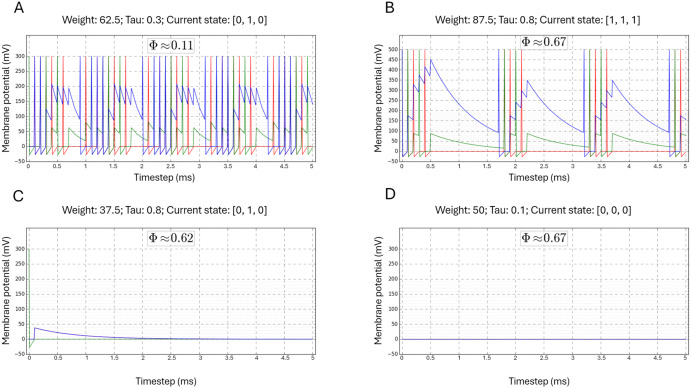
Simulation results of the IAF network. Each plot shows the evolution of the membrane potential of OR (red color), AND (green color), and XOR (blue color) IAF neurons. The IAF neuron parameters and their state are given above each graph and the Φ value is shown in an inset. Each simulation is run for 50 time steps. The figure shows a lack of correlation between IAF network activity and integrated information (Φ).

Second, we inspected plots of network activity obtained for different parameters and states. The activity of an IAF neuron, described by Equation (1), is difficult to track visually, as it does not contain afterhyperpolarization and spikes, typical for electrophysiological recordings of action potentials in real neurons. To overcome this problem, the visualization of IAF membrane potential was enhanced by adding a spike after reaching a firing threshold, followed by a negative potential representing afterhyperpolarization. We should emphasize that these graphical patterns were added to membrane potential traces at the plotting stage and did not affect the simulation. By inspecting the graphs, we noted that network activity always reached a stationary state that could be either periodic (Fig 1A and 1B) or stable (Fig 1C and 1D). We also observed no correlation between the network’s activity and the Φ value. Complex network activity may lead to low and high Φ values (Fig 1A and 1B) while trivial network activity may lead to relatively high Φ values (Fig 1C and 1D).

### Parameter sensitivity analysis

**Fig 2:**
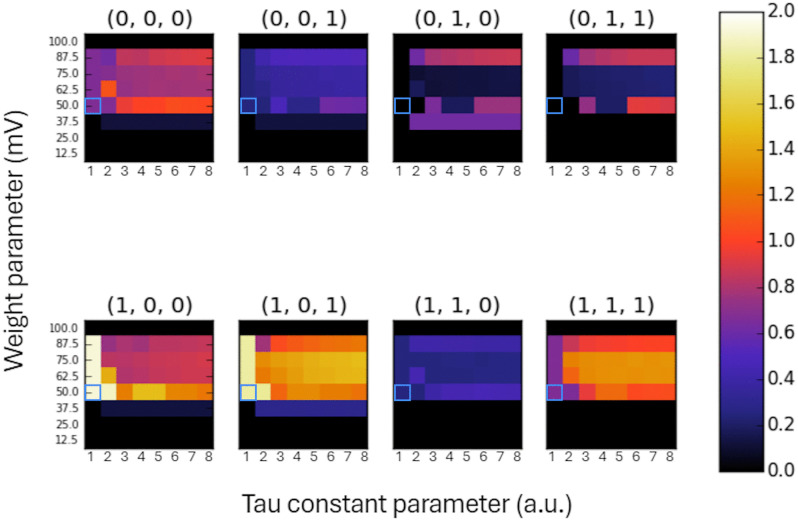
Color-coded Φ values calculated for each IAF network state, for a range of *T* and *w*_*i*_ parameters. Weights are expressed in mV, and time constants are specified in multiples of a single time-step duration (*Δt*). For the parameter set of *τ* = 1*Δt* and *w*_*i*_ = 50 mV, marked with a blue square, the IAF neurons emulate OR, AND, and XOR logic gates with the corresponding Φ values provided in Table 1. The color bar is shown at right.

Results of systematic variation of the membrane time constant and the connection weights parameters are shown in Fig 2. The results are presented as color-coded matrices of Φ values for each current state of the IAF network. For the parameter set *τ* = 1*Δt*, *w*_*i*_ = 50 mV, the IAF neurons implement the logic gates from the ABC system example provided in [12]. Φ calculation results obtained with these parameter values are marked with a blue square in each matrix and are listed in the third column of Table 1. A visual inspection of the color matrices in Fig 2 yields several general observations. (1) For the lowest (12,5 mV, 25 mV) and highest (100 mV) weight values, the Φ values are zero. (2) For the intermediate weight values, i.e., *w*_*i*_ in the range of 37.5-87.5 mV, Φ attains mostly non-zero values. Accordingly, this range can be considered the ‘working range’ of the network. (3) For each state, for *τ* = 1*Δt* and *w*_*i*_ in the range 50-87.5 mV, all Φ values are constant. (4) Depending on the weight value and the network state, the Φ values increased monotonically for increasing values of *τ* (e.g., *w*_i_ = 87.5 mV, states (0,1,0), (0,1,1)) or didn’t show a clear trend (e.g., *w*_i_ = 50 mV, states (1,0,0), (1,0,1)).

**Fig 3:**
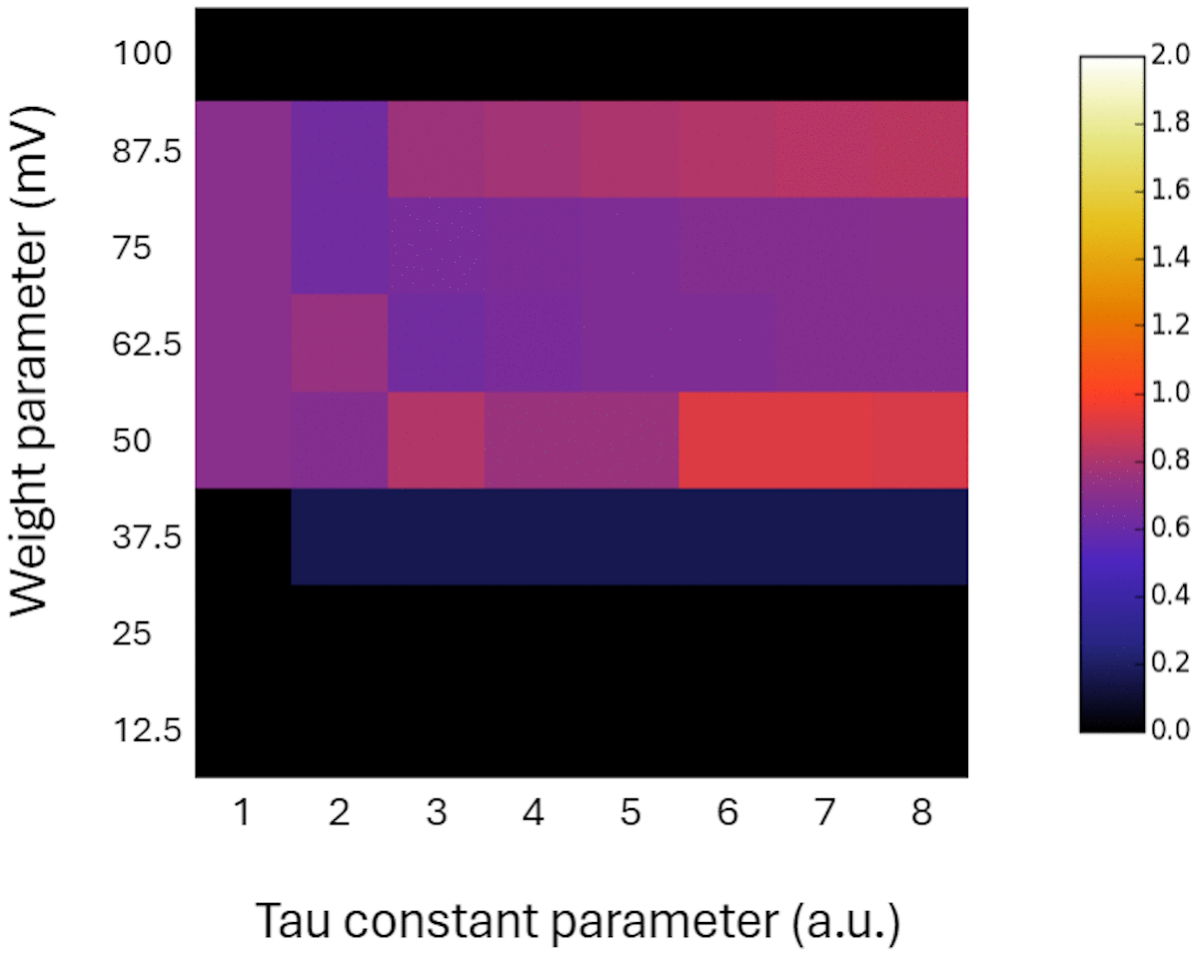
Color-coded mean Φ values across all IAF network states, for a range of *T* and *w*_*i*_ parameters. Weights are expressed in mV, and time constants are specified in multiples of a single time-step duration (*Δt*). The color bar is shown at right.

To determine whether network parameters correlate with the level of integrated information in the IAF network, we computed a matrix of average Φ values across all states. The results are shown in Fig 3. The mean Φ values across states generally reflect the observations made for each individual state. Specifically, for *τ* = 1Δ*τ* and *w*_*i*_ in the range of 50-87.5 mV, all Φ values stay constant, and for *w*_*i*_ in the range of 37.5-87.5 mV, the Φ values are mostly non-zero. However, the plot of mean Φ values shows an increasing trend in Φ as *τ* increases, especially when *τ* > 2*Δt* and *w*_*i*_ falls within the range of 62.5-87.5 mV.

### The role of random input

Many *in vivo* and *in vitro* experiments show irregular firing patterns of cortical neurons [21,22]. To investigate the influence of fluctuations on the Φ values in the IAF network, we added a stochastic process to one of the IAF neurons. It was connected to the IAF neuron with OR gate characteristics, i.e., having a spike threshold of *V*_*m*_ ≥ 50 mV (see Table 2). The stochastic signal represented a homogeneous Poisson process consisting of two states, ‘0’ and ‘1’, with state ‘1’ corresponding to spike firing. Spikes occurred independently and stochastically at a constant rate λ*.* The tested mean spike interval 1/λ was in the range 2*Δt* – 33*Δt*. Due to the stochastic nature of the simulations, the simulation duration was increased from 200 to 1000 simulation steps.

**Table 2:** Spike thresholds of the IAF neurons emulating logic gates. Activation conditions are specified for 2-input and 3-input IAF cells (second and third column, respectively). Other parameter values for logic gate emulation are: all weights *w*_*i*_ = 50 mV, *V*_*rest*_ = 0 mV, and *τ* = *Δt*.

	Activation condition	
Gate type	2-input IAF cell	3-input IAF cell
OR	*V*_*m*_ ≥ 50 mV	*V*_*m*_ ≥ 50 mV
AND	*V*_*m*_ ≥ 100 mV	*V*_*m*_ ≥ 150 mV
XOR	50 mV *≤ V*_*m*_ *<* 100 mV	50 mV *≤ V*_*m*_ *<* 100 mV

The network with stochastic input was analyzed in two ways. In the first approach, the stochastic input was treated as a fourth network element, forming a feedforward connection to the ‘OR’ IAF neuron. This situation might correspond to external fluctuating input. Accordingly, for the network with 4 binary elements, the Connectivity Matrix (CM) had 4^2^ possible connections d TMP matrix had 2^4^ states, respectively. In this system, the Φ values were always zero, regardless of other parameter values.

**Fig 4:**
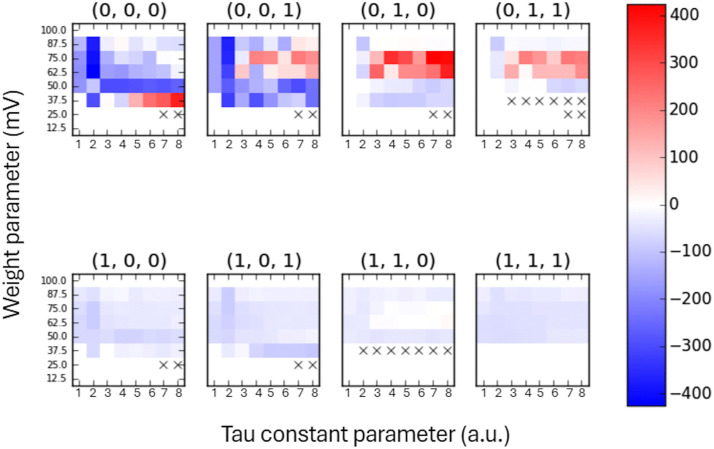
Relative change in Φ values for each IAF network state, without and with an internal noise source, calculated for a range of *T*, *w*i parameters and a mean Poisson interval 3Δ*t.* The color bar, in %, is shown at right. In each matrix, ‘x’ denotes elements for which the denominator is zero and the relative difference goes to infinity.

In a second approach, the stochastic input modified the ‘OR’ IAF neuron state but the network was still considered to have only 3 elements and the CM had 3^2^ possible connections and TMP matrix had 2^3^ states as before. This situation might correspond to intrinsic noise sources at the level of individual neurons [23]. In the presence of intrinsic noise, the Φ values were altered. The change in Φ values compared to the original values is shown in Fig 4, for a specific Poisson input rate. In this figure, the mean Poisson spike interval was 3*Δt,* meaning that a random spike was delivered to the ‘OR’ IAF neuron every 3 simulation steps, on average. It can be observed that the Φ values may increase or decrease depending on network parameters, although decreases were more frequent. For the specified value of the Poisson input rate, the maximal relative increase in the Φ value was 420%. However, for many states (all matrices in the lower row), the differences were lower than 100%. One striking feature of these results is that some parameters make the network more vulnerable to fluctuations than others regarding the integrated information measure. For states (0, 0, 0) and (0, 0, 1) for *τ* = *2*Δ*t* and *w*_*i*_ in the working range, the Φ values are significantly reduced. In other states, the drop in Φ is less pronounced but is still present. On the other hand, in the states (0, 0, 1), (0, 1, 0), and (0, 1, 1) for a weight range of 62.5-75 mV, the Φ values predominantly increased.

**Fig 5:**
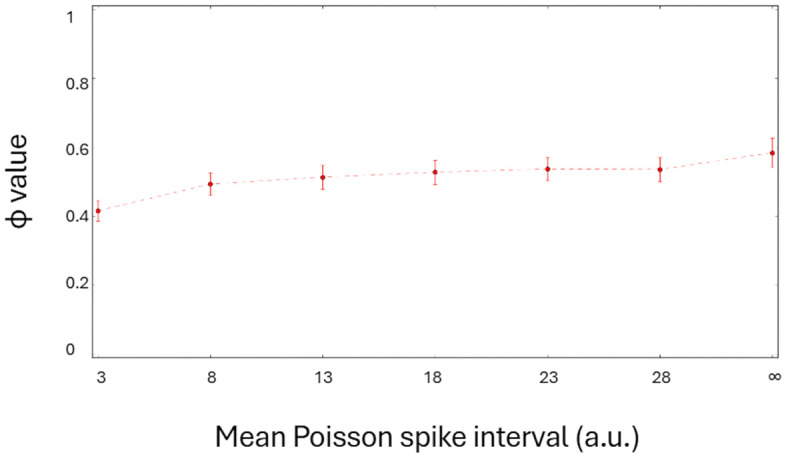
Mean Φ value across *T*, *w*i parameters and all network states as a function of mean Poisson spike interval expressed in multiples of simulation step Δ*t.* An infinite Poisson spike interval (*inf*) corresponds to a noise-free network. Error bars represent the standard error of the mean.

As described above, the noise rate, together with network parameters and states, has an irregular influence on the network’s Φ values. To illustrate the overall effect of the Poisson noise rate, we plotted the mean of all Φ values across all parameters and states as a function of the mean Poisson spike interval. The results are shown in Fig 5. The dependence of the mean Φ value on the Poisson noise rate is not pronounced. There is a weak increasing trend in the mean Φ values that reaches the mean Φ value observed without the noise (infinite Poisson spike interval), but the overall difference in mean Φ value with and without the noise is small.

## Discussion

In this study, we applied the IIT formalism to evaluate a degree of integrated information, called Φ, within a small network of integrate and fire neurons. We first showed that the Φ values of a network of IAF neurons emulating logic gates closely match the results for the OR-XOR-AND example system described in [12] (Table 1). It provided confidence that our pipeline of Φ calculation is accurate. Next, we considered a more realistic neuronal network consisting of leaky IAF neurons. We showed that the degree of integrated information depends on both the IAF membrane time constant and the connection weight parameters. The lowest and highest values of connection weights, i.e., 0–25 mV and 100 mV, respectively, lead to zero Φ values. For the lowest weight values, the network elements do not interact, meaning that the network can be decomposed into independent parts. Such a system doesn’t have a cause-effect structure and does not integrate information. For the highest weight value, the network activity for all states, except the state (0, 0, 0), is in maximal excitation conditions, i.e., ‘OR’ and ‘AND’ neurons are firing every step or every second step, while ‘XOR’ neuron fires in synchrony with ‘OR’ and ‘AND’ neurons or is saturated above the threshold and remains silent. Removing the ‘XOR’ neuron from the network doesn’t make a difference to the system, meaning that the system is reducible. Accordingly, it does not integrate information and has a zero Φ value. In contrast, for intermediate values of connection weights 37.5-87.5 mV, which we call the ‘working range’, and for a wide range of IAF membrane time constants, all elements are interacting and the network is characterized by non-zero Φ values. For each state, for *τ* = 1*Δt* and *w*_*i*_ in the range 50-87.5 mV all Φ values are constant. For these parameters, the IAF neurons represent logic gates from the ABC system example provided in [12]. As mentioned above, for these parameters, the Φ values obtained for the IAF network match the results obtained using the graphical interface described in [12] (Table 1). For *τ* above 1*Δt* and *w*_*i*_ within the working range, the Φ values are positive and vary depending on the *τ* and *w*_i_ parameter values and state (Fig 2). To determine if there is a common trend present across states, we calculated, for each *τ* and *w*_i_, the average Φ value across all IAF network states. This analysis showed that Φ values generally increased with increasing values of *τ* (Fig 3). The membrane time constant *τ* determines the neuron‘s integrative properties, meaning how quickly information about the previous state and inputs decays over one time step. Our analysis suggests that the integrated information of the network depends to some extent on the integrative properties of its individual elements. However, we also notice an exception to this rule, as Φ values were significantly higher for *τ* = 1*Δt* than for some larger *τ* for states (1,0,0) and (1,0,1) (Fig 2).

Real brain networks receive fluctuating inputs from other brain areas or peripheral sensory neurons. Additionally, the electrical activities of individual neurons have intrinsic noisy components [23–25]. Correspondingly, we tested the behavior of the IAF network and its Φ value under such conditions. In the first approach, to account for external fluctuating input, the Poisson noise spike input was treated as a fourth network element with a feedforward connection to one of the IAF neurons. In the second approach, to account for the internal stochastic component, the Poisson spike train was fed to one of the IAF neurons, as before, but we analyzed a three-neuron network.

In the four-element network, the input element affected one of the IAF neurons, but it had no causes in any of them. Accordingly, the four-element system with a feedforward connection was reducible to the sum of its subsystems and had zero Φ, as expected (e.g., set [ABCD] in Fig 14 in [1]). When the Poisson noise source was treated as an internal mechanism of the IAF neuron, the Φ values could both increase or decrease depending on network parameters without any clear pattern. In general, the relative variations in Φ values with and without noise were in the range from -400 to 400%. However, for some parameter settings, the Φ measure was highly susceptible to intrinsic fluctuations, e.g., *τ* = 2Δ*t* and states (0, 0, 0), (0, 1, 0) (Fig 4). We also examined whether the noise level, measured as a mean input Poisson spike rate, influenced the Φ measure. We computed the mean Φ value across *τ* and *w*_*i*_ parameters and all network states as a function of the mean Poisson spike interval (Fig 5). The results showed only a weak dependence of the mean Φ on the noise level.

### Limitations of the study

#### IIT analysis with a two-step approach.

Our approach has some limitations that should be acknowledged. In general, the IIT framework requires discretizing the variables of the continuous dynamical system into discrete states, which are then used in the transition probability matrix that specifies the system’s dynamics. The discretization resolution is not known a priori, hence, it was suggested to apply graining that leads to the highest value of Φ [2]. However, as pointed out by other authors, the calculation of the maximum of Φ over spatial and temporal grains is not feasible [26]. In our case, the temporal resolution of the simulated IAF network naturally corresponds to a single simulation time step. The IAF membrane potential values are represented by 64-bit floating-point numbers. Spanning the range between 0 and 100 takes more than 2^62^ distinct numbers [27]. It means that to specify IAF network dynamics with that resolution, one would need an exceedingly large state-transition matrix. A more practical approach involves dividing each neuron’s membrane potential into multiple bins. However, e.g., for 100 bins, the three-element IAF network would have 100^3^ states. Such a system would still be prohibitively large to treat using the IIT framework, while the computation of network dynamics using such crude graining could lead to inaccuracies. Accordingly, we adopted a two-step approach. First, we calculated IAF network activity with the high membrane voltage resolution (double precision floating point format). Next, at each simulation step, we mapped the membrane potential values of each neuron into a binary state, where state ‘1’ corresponded to a spike firing and state ‘0’ corresponded to all other membrane potential values. After such discretization, the three-element IAF network was characterized by 2^3^ states, resulting in an 8x8 TPM. Using this approach, we obtained Φ values for the IAF network representing the OR-XOR-AND system that were in good agreement with previously reported results for the corresponding logic-gate system (Table 1). However, this agreement does not confirm the validity of our post-hoc coarse-graining approach across different parameter settings. In particular, for *T* *> 1*Δ*t,* neurons exhibit subthreshold dynamics and identical binary states (e.g., ‘no spike’) may correspond to different membrane potentials, which could lead to different future behavior, thus potentially violating the Markovian property. On the other hand, it has been shown that spike times in a noisy network of leaky integrate and fire neurons with instantaneous synaptic integration can be described by a Markov process [28]. Hence, the specific conditions under which our approach is valid remain to be determined in future work. Further progress in this direction may benefit from the development of statistical methods to estimate transition probabilities in the IAF neurons [29] and ongoing extensions as IIT evolves to account for systems with continuous state spaces [30–32].

#### Small network size.

Another limitation is the use of only three neurons in the simulated network. Such a small system may be prone to synchronization or trivial periodic dynamics, which could affect the calculated Φ values. Indeed, our three-neuron system exhibited trivial activity under certain conditions. (i) For a coupling weight of 100 mV and all values of *τ*, the network converged within a single step to either a synchronized, periodic behavior (e.g., (1,1,0) → (1,1,0)) or an alternating, periodic firing pattern (e.g., (0,1,0) → (1,0,0) → (0,1,0)). (ii) For weights of 37.5 mV or lower, across all *τ* values, network activity decayed and ceased within a few steps (see Fig 1C for an example). (iii) For the initial state (0,0,0), no activity was observed for any parameter combination (see Fig 1D). Indeed, for trivial activity, i.e., for the lowest (37.5 mV or less) and highest (100 mV) weight values, the Φ values are zero or very low (Fig 2). However, for the weights in the range 50–87.5 mV, the activity is more complex (see Fig 1AB for an example) and the Φ values remain non-zero. It shows that, across a wide range of connectivity parameters, even a three-neuron network can generate meaningful, i.e., non-trivial, dynamics and integrate information. We should also note that even a trivial activity in one state doesn’t exclude integration of information in that state. E.g., in the state (0,0,0), the network activity makes transitions (000) → (000), yet the Φ values are non-zero (Fig 1D and Fig 2). This is because even though IIT evaluates Φ at a particular current state, it formally compares the actual state to alternative states, and the difference indicates the causal effect.

#### Biological realism of the ‘XOR’ neuron.

Still another limitation concerns setting a firing threshold range in the ‘XOR’ IAF neuron, as shown in the Methods section (Table 2). This choice was motivated by an effort to implement the OR-XOR-AND system using the IAF network to compare our results with those in the literature [12]. It may seem unrealistic, as biological neurons are typically characterized by a single potential threshold instead of a range of thresholds. Accordingly, they have been thought to perform AND and OR but not XOR operations [33]. However, recent findings indicate that calcium-mediated dendritic action potentials allow biological human neurons to execute the XOR operation as well [34], providing physiological support for our parameter setting.

#### External noise source considered as an internal mechanism.

An additional limitation relates to random spikes applied to one of the IAF neurons in our three-neuron system. In such a case, the external noise source should be considered a background condition. However, in IIT 3.0, background conditions are inherently fixed external constraints [1], whereas our stochastic spike source follows a Poisson process. To address this constraint, we treated the noise source as an internal mechanism of the IAF neuron, which may reflect stochastic membrane channel dynamics leading to spontaneous action potentials [23]. We also note that the results with added noise are preliminary, as they were obtained by adding random spikes only to the ‘OR’ IAF neuron. Applying the noise to other neurons could have different effects. Additionally, to determine if the noise-related conclusions are valid, they should be tested across a wider range of systems. Nonetheless, our approach might not be a viable solution since IIT 4.0 addresses the limitations of IIT 3.0 by assuming that background units do not need to be deterministic [35].

#### Integrate and fire vs Hodgkin-Huxley neurons.

Analysing Φ of the IAF neuronal network might be considered another limitation, since the Hodgkin–Huxley (HH) model seems to be the most natural choice for applying the IIT formulation to a realistic neurobiological system. The IAF neurons have a well-defined threshold, can be formulated as a discrete-state system, and are Markovian. On the other hand, biophysical neuron models (e.g., Hodgkin–Huxley), unlike IAF models, have continuous state variables and lack a threshold-voltage parameter. Furthermore, in the classic Hodgkin-Huxley equations, the voltage dynamics depend on gating variables (m, h, n) that represent the probabilities of channel activation and inactivation. Synaptic currents require additional synaptic conductance variables (g_syn). To preserve the Markov property, the full state of each neuron (V, m, h, n), together with synaptic variables, must be considered. With a single synaptic variable per neuron, the three-HH neuron system would consist of 3x5 = 15 variables. Even for binary discretization of each variable, it would result in 2^15 possible states and the TMP size of 2^15 x 2^15, which is much larger than the TMP size for a three-IAF neuron network.

Alternatively, if one treats the membrane potential variable as binary (spiking/not spiking at each time step) and neglects all other variables, the three-HH neuron system would have only 2^3 = 8 possible states. However, with this simplification, the system loses all subthreshold dynamics and the Markovian property, since, for a given V, the gating variables can be in different configurations. Although a similar problem arises for IAF neurons with binary states, spike times in networks of IAF neurons can be described by a Markov process, unlike in conductance-based models [28]. It demonstrates that applying IIT to realistic neural models, such as the Hodgkin-Huxley model, is more challenging than applying it to IAF neurons.

Finally, it is important to acknowledge the limitations of generalizing conclusions to other systems. For example, findings related to the time constant and noise may be specific to the analyzed network implementing the OR-AND-XOR system. To assess their generalizability, a more comprehensive analysis of diverse systems is required.

## Conclusions

The main contribution of this study is several empirical findings regarding the application of Integrated Information Theory to artificial integrate-and-fire neuronal network. They may have implications for understanding consciousness through the lens of IIT. First, we found no relationship between IAF network activity and Φ values, indicating that Φ cannot be reliably estimated from the time evolution of spontaneous brain activity. Second, random perturbations applied to the network could either increase or decrease Φ values but averaging across network parameters and states revealed only a weak dependence on noise magnitude. This suggests that IIT 3.0 primarily considers fully deterministic systems, unlike the inherently noisy human brain. Notably, IIT 4.0 has begun to address this limitation by incorporating the degree of noise and determinism in the binary unit activity [35]. Third, when random perturbations were treated as external input to the network, the system had a zero Φ value. It suggests that the IIT theory focuses on systems with activity that arises intrinsically, thus corresponding to states such as sleep and dreaming, while excluding considerations of external inputs that characterize an awake brain receiving sensory input from the environment.

In summary, our study shows a potential application of IIT in a real neuronal system. It also reveals that artificial neuronal networks can integrate information and have non-zero Φ values. However, this does not necessarily imply that such networks are conscious. As Tononi and Koch argue [2], consciousness is an intrinsic property of physical systems and cannot exist within virtual or simulated environments.

## Methods

### PyPhi module

To compute the Φ value of a network, we used PyPhi Python library created by the IIT developers [36]. In the PyPhi library, a network is specified using a Transition Probability Matrix (TPM) and a Connectivity Matrix (CM). TPM contains the system’s transition probabilities between any two states. CM is a square binary matrix indicating how the nodes of a system are connected. For a network specified by TPM, CM, and a network’s state, we used pyphi.compute.phi() function to compute its Φ.

### Φ of the logic gate network

To use the PyPhi library we developed custom scripts to generate CM and TPM of a network. To test these scripts, we simulated a mutually connected OR-AND-XOR system of logic gates identical to the ABC system analyzed in [1]. For each specific current state, TPM was computed using eight iterations, which was enough to cover all possible transitions in the network. At every iteration, the previous (at *τ*) and next (at *τ* + 1) binary states were noted and a probability distribution of the next state as a function of the previous state was calculated. The simulation was repeated eight times, starting once with each possible current state of the system. At the end of the whole simulation process, each distribution was normalized and put together into the ‘empirical’ TPM. As expected for the deterministic, logic gates system, the values in the TPM were 0 or 1. By feeding the PyPhi toolbox with simulated TPM and CM and different network states, we obtained Φ values identical to the ones described in the original publication (Table 1), confirming that our simulation environment was working correctly.

### Integrate and Fire neuron

The implementation of the IAF neuron was based on the Matlab code of Practical 5 from the Neural Computation course [37]. The value of the IAF neuron membrane potential in mV, at time *τ*, was calculated with the following equation:


$$\begin{array}{c} V_{m}(t)=V_{m}(t-1)+\frac{\Delta t}{\tau }\left(V_{rest}-V_{m}(t-1)\right)+\sum_{i}^{n}w_{i}{Input}_{i}(t-1) \end{array}$$ (1)

where: *V*_*m*_(*τ* - 1) – membrane potential at previous time-step, *V*_*rest*_ – resting potential (mV), *Δt* – timestep duration (ms), *τ* – membrane time constant (ms), *w*_*i*_ – connection weight from the *i*th neuron (mV), *Input*_*i*_ – input value of the *i*th neuron (dimensionless), *n* – number of neurons.

The IAF neuron evolves at discrete time steps until the membrane potential reaches a threshold *V*_*th*_, which leads to the IAF neuron spike firing and resetting the membrane voltage to its resting potential. At the next time step, the IAF neuron resumes integration of its inputs. We note that Equation (1) represents the so-called leaky integrate and fire neuron as its membrane potential decays exponentially in the absence of input. It is noteworthy that the system, made up of elements defined by Equation (1), satisfies the Markov condition required for IIT analysis.

### Logic gate network with IAF neurons

Setting the membrane time constant equal to a single time-step leads to simplified IAF neuron dynamics described by the equation:


$$\begin{array}{c} V_{m}(t)=V_{rest}+\sum_{i}^{n}w_{i}{Input}_{i}(t-1) \end{array}$$ (2)

Under these conditions, selecting appropriate values of *w*_*i*_ and *V*_*th*_ allows individual IAF neurons to act as logic gates. Hence, as a first step toward the transition from the logic gate network to the IAF network, we recreated the OR-AND-XOR system of logic gates described in [1], using three connected IAF neurons. In such a network, each neuron receives two inputs from the other two cells. To allow simulation of external input, we also considered neurons receiving one additional input. The firing threshold values, *V*_*th*_, for the 2-input neurons and 3-input neurons, are described in Table 2. To emulate logic gates, all weights were set to 50 mV, the resting potential was set to 0 mV, and *τ* = 1*Δt*.

We computed eight simulation steps of the simplified IAF network described by Equation (2). It was sufficient to ensure that the IAF neurons entered a stationary regime, i.e., remained in the below threshold state or exhibited periodic behavior. After the simulation has finished the obtained membrane potential traces were used to calculate the Φ values. To compute the TPM, we associated state ‘1’ with spike firing of the IAF neuron and state ‘0’ with all other *V*_*m*_ values at every simulation step. In this way, we reduced multiple IAF neuron states into binary states. E.g., subthreshold membrane potential values 0 mV and 50 mV of the ‘AND’ IAF neuron were assigned the ‘0’ state. Subsequently, these binary states were used to create a probability distribution of the next network state as a function of the previous state. The simulation was repeated eight times, starting once with each possible current state of the system, e.g., (0, 1, 0) where ‘0’ corresponded to resting potential and ‘1’ corresponded to a spike firing. At the end of the TPM calculation, each distribution was normalized.

### Network of IAF neurons

In simplified IAF neurons described by Equation (2), the membrane potential values depend only on the resting potential and inputs in the previous time step. This differs from real neurons, which integrate previous inputs with previous membrane potential values. Accordingly, to simulate a network of leaky IAF neurons that more closely resemble real neurons, we used Equation (1) with the membrane time constant *τ* not restricted to a single time step. We also investigated a range of weight parameters to see if connectivity strength influenced the information integrative properties of the network. The *τ* parameter was varied from 1*Δt* to 8*Δt* in steps of *Δt* and the *w*_*i*_ parameter was changed from 0 to 100 mV in steps of 12.5 mV. The number of simulation steps was increased to 200, with membrane potential graphs closely inspected, to ensure that for each current state, the future behavior of neurons reached a stationary regime. For TPM calculations, it was assumed, as before, that the state of the IAF neuron was ‘1’ at the time-step of spike firing and was ‘0’ otherwise. At the end of the TPM calculation process, each distribution was normalized. Despite the system being deterministic, the TPM contained non-integer values between 0 and 1. For each parameter set, the simulation was repeated eight times, once for each possible current state of the system. Next, the transition probabilities were combined into a single TPM.
